# Predicting proprioceptive cortical anatomy and neural coding with topographic autoencoders

**DOI:** 10.1371/journal.pcbi.1012614

**Published:** 2024-12-04

**Authors:** Max Grogan, Kyle P. Blum, Yufei Wu, J. Alex Harston, Lee E. Miller, A. Aldo Faisal

**Affiliations:** 1 Department of Bioengineering, Imperial College London, London, United Kingdom; 2 Department of Physiology, Northwestern University, Illinois, United States of America; 3 Institute of Artificial & Human Intelligence, University of Bayreuth, Bayreuth, Germany; Brown University, UNITED STATES OF AMERICA

## Abstract

Proprioception is one of the least understood senses, yet fundamental for the control of movement. Even basic questions of how limb pose is represented in the somatosensory cortex are unclear. We developed a topographic variational autoencoder with lateral connectivity (topo-VAE) to compute a putative cortical map from a large set of natural movement data. Although not fitted to neural data, our model reproduces two sets of observations from monkey centre-out reaching: 1. The shape and velocity dependence of proprioceptive receptive fields in hand-centered coordinates despite the model having no knowledge of arm kinematics or hand coordinate systems. 2. The distribution of neuronal preferred directions (PDs) recorded from multi-electrode arrays. The model makes several testable predictions: 1. Encoding across the cortex has a blob-and-pinwheel-type geometry of PDs. 2. Few neurons will encode just a single joint. Our model provides a principled basis for understanding of sensorimotor representations, and the theoretical basis of neural manifolds, with applications to the restoration of sensory feedback in brain-computer interfaces and the control of humanoid robots.

## Author summary

It is well established that proprioception is essential for effective for motor control, yet our understanding of proprioceptive coding in somatosensory cortex lags behind that of more studied sensory modalities such as vision and touch. Here, we use unsupervised learning of a deep neural network imposed with biological constraints to reproduce coding properties of proprioceptive neurons in area 2 of primary somatosensory cortex. With this model, we demonstrate that the tendency for area 2 neurons in close physical proximity to share similar directional tuning can be explained by local topographic organisation driven by Mexican hat lateral effects, a phenomenon that is otherwise unobservable with the spatial resolution of available electrode microarray recordings. This provides, to the best of our knowledge, the first evidence of local topographic organisation in area 2 proprioceptive neurons. We also predict that the structure of proprioceptive receptive fields is typically multi-joint rather than single-joint in nature and provides, to the best of our knowledge. We find that our model best reproduces the tuning properties of neural data when training on natural kinematic recordings, rather than those from a stereotyped task, underlining the importance of training on data that reflect the natural distribution of sensory stimuli.

## Introduction

Somatosensation includes the familiar sense of touch, provided by receptors in the skin, and proprioception, the much less consciously perceived sense that informs us about the pose, motion and associated forces acting on our limbs. Both are essential for our ability to plan, control and adapt movements. In engineering, the control of robotic movement would be impossible if the controller did not know the location of its actuators; correspondingly in human motor control, popular frameworks for explaining many types of movement, such as optimal feedback control theory [1,2], are dependent on feedback such as that provided by proprioception. Moreover, individuals with proprioceptive neurological deficits, such as patient IW, have profound motor deficits even in the presence of vision and an intact motor system [3,4]. Similarly, recent major developments in the control of limb movement through intracortical Brain Computer Interfaces are centred on restoring somatosensory sensory feedback as well as effecting motion [5–8]. Thus far, these efforts have been almost exclusively limited to the sense of touch, in part because of our better understanding of that modality. However, restored proprioceptive feedback is also likely to be needed to adequately restore functional capability [9–11].

Unlike hearing or vision, proprioceptive afferent pathways originate not from a single organ but from diverse families of mechanoreceptors within muscles, tendons, joints, and even the skin [12]. Proprioceptive information ascends within the dorsal column pathway through the dorsal root ganglia, the dorsal column nuclei, and thalamus before arriving in the primary somatosensory cortex (S1). Crucially, neurons are somatotopically organised throughout this pathway, meaning that neighbouring neurons encode stimuli from closely related parts of the body. This gives rise to the sensory homunculus, which results from the ordered mapping of tactile representations of the body’s surface across the cortical surface [13].

Although proprioception is generally acknowledged to be critical to motor behaviour, the corresponding proprioceptive maps ‐ particularly that of primate area 3a, but also the mixed modality area 2 ‐ are much less obviously structured than those of the touch (areas 1 and 3b). We know that the proprioceptive and tactile systems often encode overlapping information, e.g. mechanoreceptors in our skin and interosseous membranes respond to deformation and vibration, contributing to the sense of body position and movement [3]. Indeed, the firing rates of somatosensory neurons with cutaneous receptive fields in area 2 can be used to decode limb movement as accurately as those with muscle fields [14]. While proprioceptive cortical areas are critical for our ability to generate goal-directed complex behaviour it is unclear what properties of proprioceptive cortical coding facilitate this capability. Crucially, we lack an accepted hypothesis about the computational principles that drive the mapping of proprioceptive arm representations onto the cortex.

The limited nature of our understanding of proprioceptive neural representations in the brain is in large part due to the difficulty of delivering independent proprioceptive stimuli in comparison to other senses, such as vision [15] and touch [16]. To overcome this limitation, we combined computational modelling and natural movement kinematic data to test hypotheses of organisational mechanisms that are currently beyond the capability of microarray recording techniques, where the distance between electrodes is too great to observe spatial organisation at the level of neighbouring neurons. This combination of experiment and theory has been useful for explaining population-level coding in other sensory modalities, such as vision [17] and touch [18,19], but has only recently begun to be applied to proprioception [20,21]. Much of the computational theoretical work on other senses has either focused on predicting specific neural coding features from natural sensory statistics [18,22], or has ignored the details of neural coding and focused on the spatial distribution of stimulus representation across the cortex [23].

**Fig 1:**
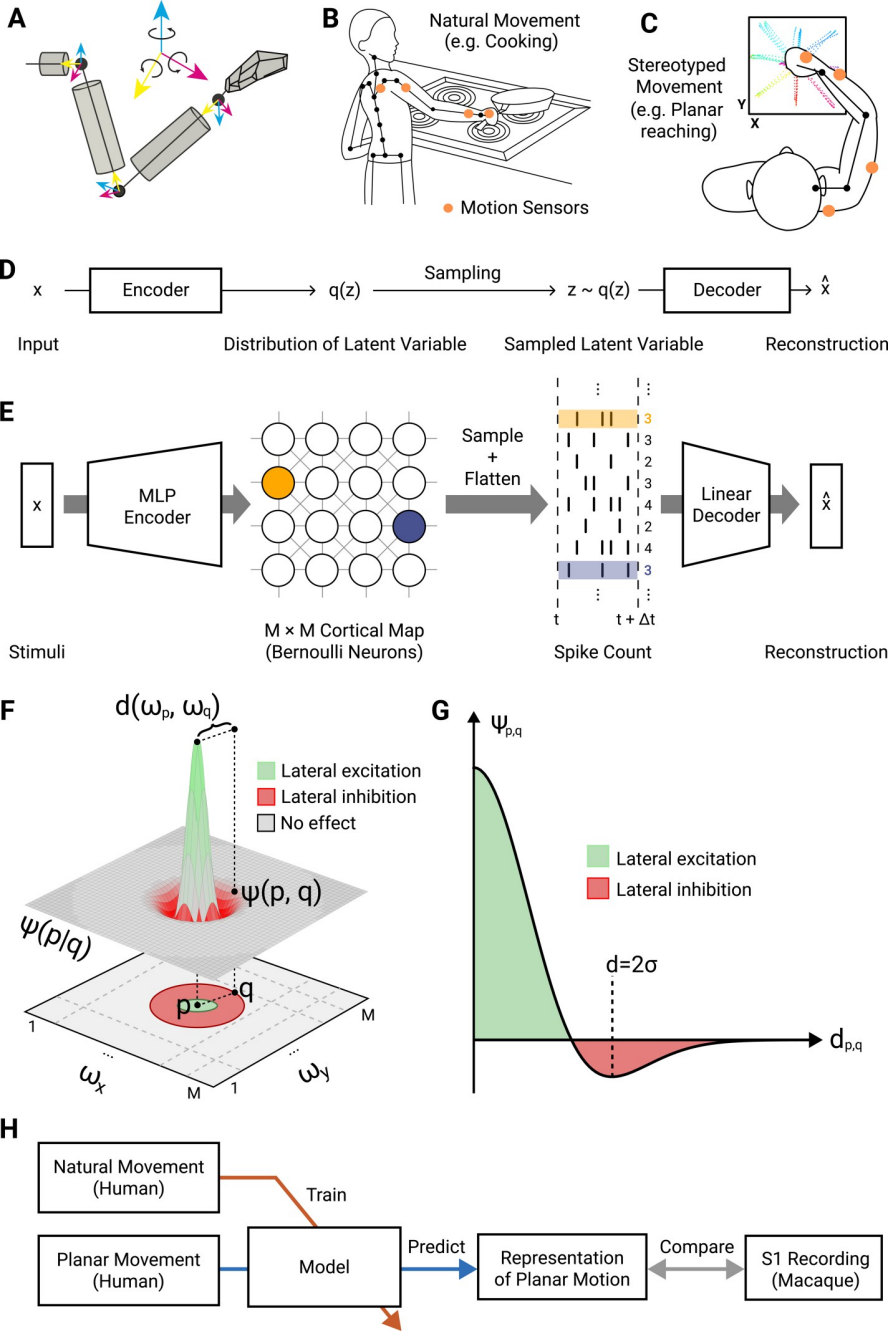
Task Context and model architecture. **(A)** 9-dimensional arm movement data acquired from motion capture suit is represented by Euler angles between body segments, following the ISB Euler angle extractions [29] in a ZXY coordinate frame. **(B)** Illustrative example of movements carried out in the natural scenario (cf. S1 Fig for further examples). **(C)** Illustrative example of planar centre-out reaching movements. **(D)** General architecture of a VAE: Inputs are encoded as a latent distribution, from which samples are decoded to reconstruct the original input. **(E)** Topo-VAE architecture. Our cortical neural representation is modelled by an 80x80 cortical grid of artificial neurons in the latent layer q(z). In contrast to conventional VAEs which model these latent neurons as multi-dimensional Gaussian random variables, we used Poisson random variables. Input stimuli drive these model neurons via a multi-layer perception (MLP) encoder network (hidden layer of 500 neurons). A linear decoder is applied to the spike counts emitted at a given timepoint (corresponding to a single input sample), to reconstruct the sensory input stimuli. **(F)** To embed the model neurons in a cortex-like topography we assign each neuron a 2D position and define their lateral interactions as a Mexican-hat function of the distance between them. **(G)** This interaction ψ(p,q) is also characterised by a length scale σ. Nearby neurons are excited, intermediate-range neurons are inhibited and there is no effect on distant neurons. See methods for further details. **(H)** Flow of natural and planar movement data (joint angular velocities) in this work.

Our chosen system was the proprioception of the right arm (Fig 1A) for which we had collected natural behavioural data from daily life in humans (food preparation, eating, etc; Figs 1B and S1) as well as constrained, planar centre-out reaching in both humans and monkeys (Fig 1C). This data describes the joint angle kinematics of the of the body over time and thus stands in for the proprioceptive sensory state. We aimed to relate kinematics to proprioceptive encoding with modelling and comparisons to existing single-unit recordings from monkeys. We formulated a novel computational model that predicts both neural coding of single neurons and the spatial organisation of these neurons across the cortex (Fig 1D–1G). We chose a small set of general computational elements that reflect principles and mechanisms found in sensory systems but have not been previously unified, which are as follows: Models should 1) use the information maximisation principle, which postulates that efficient sensory representations in the brain reflect natural sensory statistics, and implies that they are essential in shaping neural representations, 2) be stochastic, generative, and decodable, to reflect the natural variability in the data, produce neural activity to mimic that of the biological system, and offer the means to reconstruct the relevant original sensory input from its output, 3) implement, in Marr’s sense [24], neural computations that are performed by locally interacting neurons through synaptic interactions, rather than by an abstract computational machine.

In the context of proprioception, these principles require that our model should learn how to translate proprioceptive inputs from movements of the body into a latent representation of proprioception (which is read out as neural firing). In addition to performing efficient feature learning of a spike-based code, the model should have minimal inductive bias regarding a particular sensory modality, in that it can be generalised to modelling other regions of sensory cortex. By training the model on natural movement stimuli, it should learn representations that can maximise information about the environment with the limited bandwidth of neurons that is available at any given encoding step, thereby implementing efficient coding [25–27]. Given these requirements, we chose to use an unsupervised deep neural network model, a variational autoencoder (VAE) [28] (Fig 1D) to perform efficient feature learning of proprioceptive stimuli. The primary training objective of an autoencoder is to reconstruct the input stimuli, enabling the model to learn in an unsupervised manner. Furthermore, in our use case, the encoder component of the VAE model parameterises a latent Bernoulli distribution to imitate the activity of spiking neurons.

In following our third outlined principle and the observation that spatial organization is relevant to cortical coding, we introduce a 2-dimensional structure to the latent (“bottleneck”) layer of the VAE (Fig 1E). A conventional “vanilla” VAE model will capture the properties of encoded sensory features but would be devoid of any of the spatial properties of cortical neurons that are critical for determining how sensory stimuli are represented across the cortical surface. Incorporating spatial relationships in neural coding models has proven an important consideration for understanding neural computation, where anatomical structure and function in neural computation are fundamentally linked by biophysical constraints [30–33]. We therefore also implemented a topographical mechanism, using a lateral interaction term between the units in the VAE’s latent layer (see Methods for detailed motivation and mathematical description; Fig 1F). This term corresponds biologically to a spatial distribution of short-range excitatory and longer-range inhibitory synaptic interactions between neurons (Fig 1G).

We call our model the “topo-VAE” and compared it with existing recordings from area 2 of somatosensory cortex. It could equally well be applied to other proprioceptive areas such as 3a or even 5. With further adjustments, such as replacing the linear decoder (or even the MLP encoder) layer with more elaborate architectures, it should be possible to apply this model to the study of spatial organisation in other sensory modalities, including touch, vision, and hearing. Here, the approach has enabled us to uncover novel insights into proprioceptive coding by linking anatomical structure and function so that we can test our model’s predictions using the sparse set of obtainable data.

## Results

We developed a novel model of cortical sensory representation (see Methods for details) that predicts both function and structure–the topo-VAE model. We trained, and validated, and tested our model as follows (Fig 1H): We trained our topo-VAE model on natural daily human arm movement kinematics and explored the emergent proprioceptive representations in its cortical layer (i.e. the latent layer of the topo-VAE). After training, we used the model to generate neural responses by providing it kinematic data from a centre-out reaching task performed by human subjects. We then compared the properties of these simulated neural activities (i.e. the generated spike trains) to those of S1 neurons which were previously recorded from monkeys performing a planar centre-out reaching task, for which macaque and human arm kinematics are known to be similar [34]. We characterised the proprioceptive neural representations at two levels of description: first, we considered the spatial tuning curves of individual neurons and second, we considered the organisation of tuning preference maps across our model’s cortical surface.

**Fig 2:**
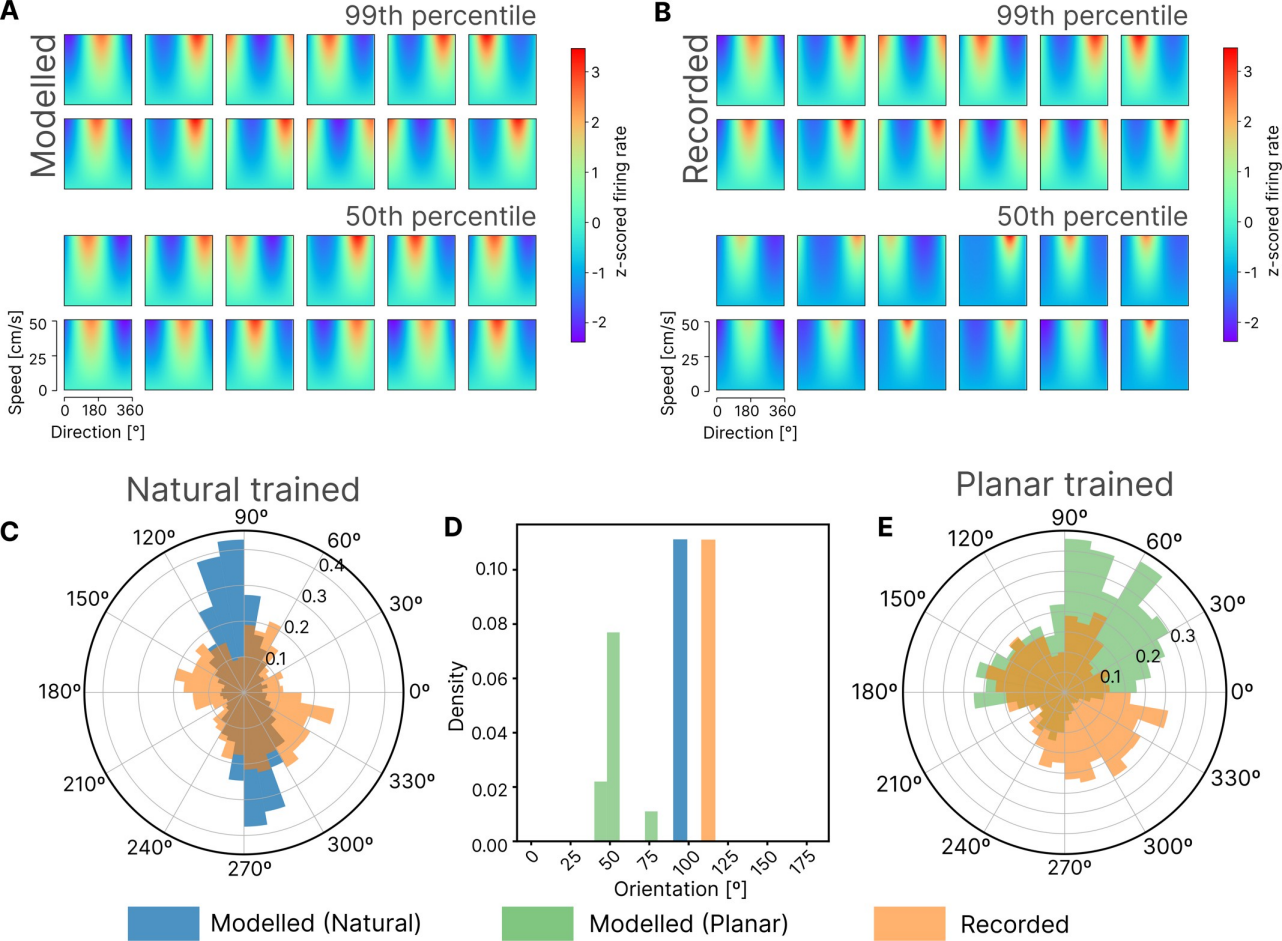
Comparing tuning of modelled and recorded neurons. (**A)** Tuning maps from example neurons observed in the latent layer of the topo-VAE under planar movements **(B)** Tuning maps of example area 2 neurons during planar movements, compared to their closest matches in A. Top panels are examples of the highest quality matches, bottom panels are examples from the 50^th^ percentile of match quality. (**C)** Circular density histograms of preferred directions in our topo-VAE model trained on natural data (n = 6400) (blue) and neurons recorded from area 2 of 3 monkeys (n = 383) (orange). Note, that the neural models were not fitted to monkey neural data, but directly predicted from the statistics of natural human body kinematics. **(D)** Frequency histogram of mode bias in PD distributions for topo-VAE models trained on natural data (blue) and stereotyped centre-out reach data (n = 10 for each condition) vs mode bias of PD distribution in recorded neurons (Orange line). **(E)** Circular density histograms of preferred directions in our topo-VAE trained on stereotyped centre-out reach data (n = 6400) (green) and neurons recorded from area 2 of 3 monkeys (n = 383) (orange).

We used three sets of measurements to summarise the neural coding properties of S1 neurons for this centre-out task: 1) the preferred direction (PD) of single-neuron firing rates, 2) features of the tuning surface defined by firing rate modulation as a function of both direction and speed of movement, and 3) the overall distribution of firing rates throughout the behaviour. First, we analysed PDs of neurons in the recordings and in our modelling (Fig 2). The PD refers to the movement direction of the hand (when performing the centre-out reaching task) in which a neuron was most active. The tuning surface refers to the inferred firing rate response of a neuron to a given range of endpoint directions and velocities, which is generated using a Poisson GLM trained to map from x and y endpoint velocities to the firing rate of a neuron. We found that generally, the parametrically fit PD tuning surfaces exhibited by modelled neurons were similar in appearance to those of recorded neurons (cf. Fig 2A and 2B, top panel for the 12 best matches between modelled and recorded neurons, bottom panel for 12 examples from the ∼50^th^ percentile of match quality. Further examples of each can be found in S2 Fig). At the population level, while there was a statistically significant difference (p<0.005; Kolmogorov-Smirnov test) between the distribution of PDs of modelled and recorded neurons (Fig 2C), the distributions were similar in that they were both bimodal (S3 Fig), with a bias towards the 120°/300° reach axis (Mean: 97° vs 115°, natural-trained model vs recorded) (Fig 2D). Previous experimental studies have suggested such coding biases are a natural consequence of musculoskeletal constraints in the arm [35,36], which cause the natural distribution of right arm reach movements to favour the 120°/300° reach axis. Under the efficient coding hypothesis, this becomes reflected in the coding properties of the sensory neurons. To test this theory, we trained a second model on stereotyped movement stimuli from the centre-out reach task only. We compared the reconstruction quality (R^2^ scores) of natural-trained and planar-trained models by evaluating them on planar and natural movement data, respectively. We found that the natural-trained model generalised to planar movement data significantly better than the planar-trained model generalised to natural movement data (0.81 ±0.02 vs 0.73 ±0.04, natural-trained vs planar-trained; p<0.005; Wilcoxon signed-rank test). The planar-trained model produced a PD distribution that was also significantly different from the PD distribution of recorded neurons (p<0.005; Kolmogorov-Smirnov test), but unlike the natural-trained model, the reach axis bias was perpendicular to that of recorded neurons (Mean: 49° vs 115°, planar-trained modelled vs recorded) (Fig 2D and 2E). This suggests that certain features of proprioceptive coding are only emergent when the coding is conditioned on the natural distribution of stimuli.

**Fig 3:**
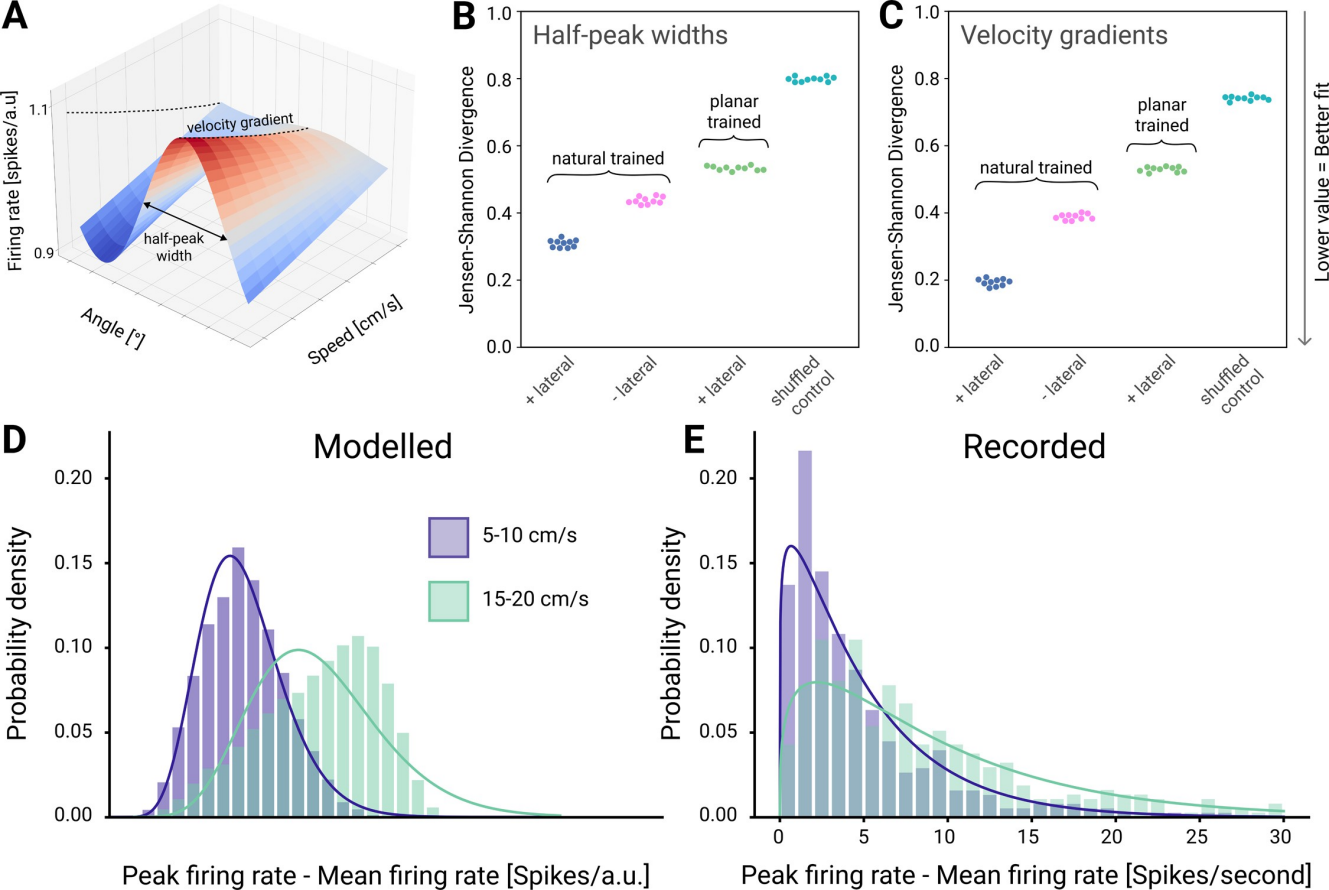
Comparing tuning surface features and firing rate distributions between modelled and recorded neurons. (**A)** Tuning surface from a single modelled neuron, illustrating two measures we computed to summarise neuronal modulation with velocity: half-peak width of spatial tuning (at maximum end-point speed) and velocity gradient. (**B)** Jensen-Shannon divergence between the distributions of half-peak widths in modelled neurons trained under different conditions (with (+) and without (-) lateral effects, trained on natural data, trained on planar data) (6400 neurons, 10 models with different weight initialisations per condition) and recorded neurons (383 neurons). The shuffled control is the same as the natural trained model with lateral effects, except the neural activity is shuffled prior to training the Poisson-GLM in the tuning surface calculation step. **(C)** Equivalent plot to (B) for velocity gradient distributions. (**D)** Firing rate modulation (peak rate minus mean rate) by endpoint-velocity across all reach directions in modelled (6400 neurons) aI(**E)** in recorded neurons (383 neurons). Histograms represent actual distributions, whereas lines represent fitted gamma distributions (R^2^>0.95 for all; Kolmogorov-Smirnov test).

While preferred direction is an informative neural correlate, we looked to better compare tuning properties by extracting geometric features from the tuning surfaces of modelled and recorded neurons and evaluating how different model properties affect similarity. Two such features were compared (Fig 3A): Half-peak widths measure how sharp the tuning of a neuron is, where smaller half-peak widths indicate sharper tuning, and velocity gradients measure how sharply the firing rate of a neuron changes as a function of the endpoint velocity in the direction of tuning (note that the velocity gradient measurements were adjusted to control for differences in mean firing rate between modelled and recorded neurons). While there was a statistically significant difference between the feature distributions of modelled and recorded neurons (p<0.005 for both features and all model variations; Kolmogorov-Smirnov test), Jensen-Shannon divergence between distributions was minimised (Fig 3B and 3C) when the model included lateral effects and was trained on natural movement data. This would suggest that both model constraints are important for reproducing the tuning properties of recorded neurons. One concern was that the distributions of tuning curve geometry between modelled and recorded neuron populations were similar only because of the Poisson GLM fitting process–perhaps fitting a Poisson GLM to any random data would produce distributions like these. To verify that this was not the case, we shuffled the firing rate data from modelled neurons to remove any correlation between endpoint velocities and firing rates and trained an additional Poisson GLM on this data. The shuffling produced a very different pair of distributions for the geometric features, with flat, poorly tuned curves–suggesting that any similarities we saw between the original unshuffled curves were in fact due to similarities in the underlying structure of the modelled and recorded neuron data. This provides an empirical lower bound of the similarity between feature distributions, and would suggest that similarity between the distributions of modelled and recorded neurons is not trivially the result of using a GLM (Fig 3B and 3C).

Finally, we examined endpoint-velocity modulation of firing rate distributions, which were well fit by Gamma curves for both modelled (Fig 3D) and recorded neurons (Fig 3E; R^2^ > 0.95 for equal numbers of neurons). We compared the distributions at different endpoint velocities and found that they differed significantly in both modelled and recorded neurons (p<0.005; two-sided Wilcoxon signed-rank test), indicating velocity dependence in both.

In summary, modelled neurons in the topo-VAE exhibit various tuning properties such as preferred direction and endpoint-velocity modulation of firing rates which are structurally similar to those of recorded neurons. While the population statistics of these tuning properties differ significantly between modelled recorded neurons, the combined lateral effects that constrain our model and the training data that reflects the natural distribution of arm movement, increase the similarity, all without fitting our model to any neural data.

**Fig 4:**
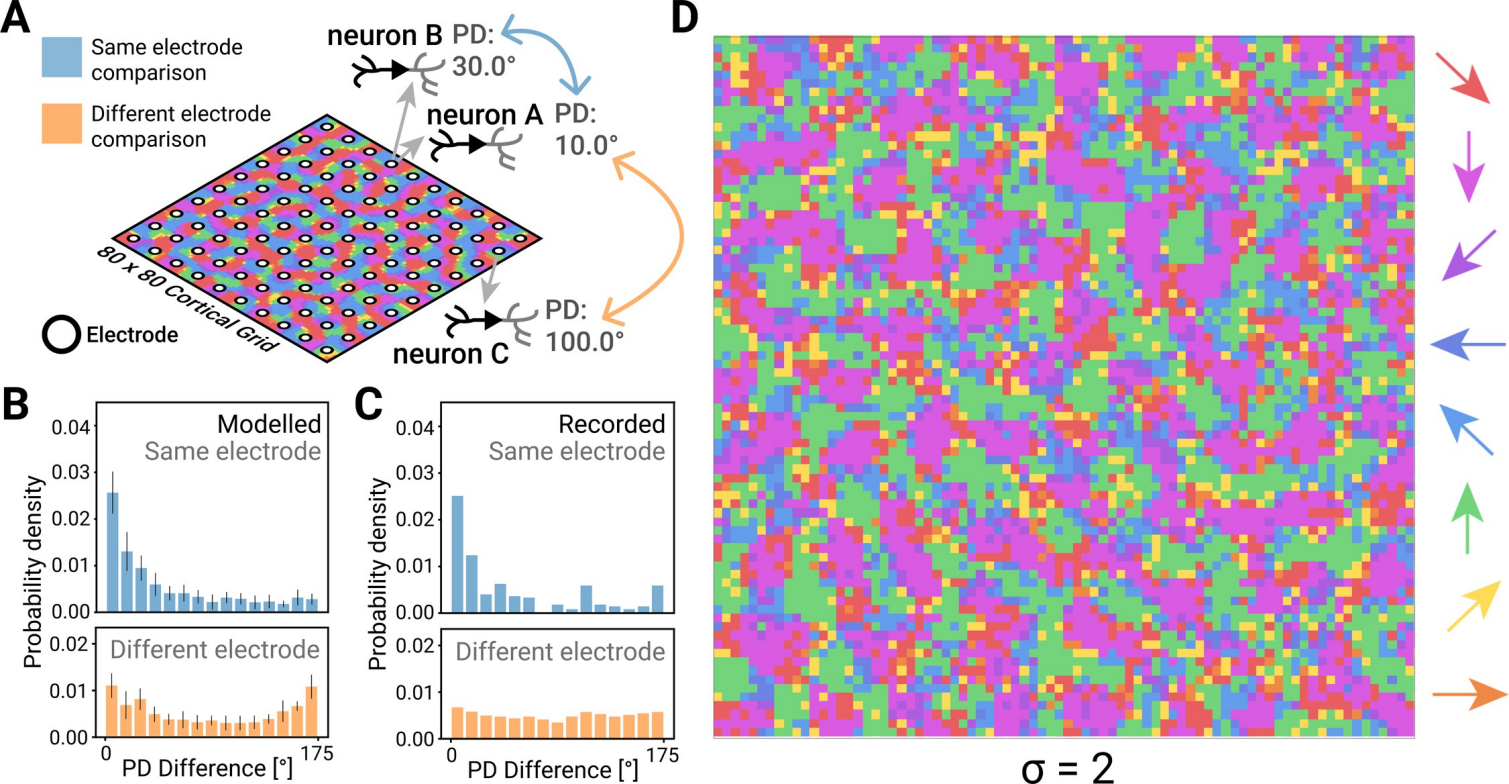
Predicting the topography of proprioceptive cortex. (**A)** Illustration of pairwise comparisons of preferred directions recorded on same (blue) and different (orange) electrodes, performed in recorded and modelled neurons. Neuron A and B are recorded from the same electrode, Neuron C is recorded from a distant electrode. **(B)** Distribution of PD difference for same electrode (blue histograms) and different electrode (orange histograms) comparisons in modelled neurons and **(C)** recorded neurons (length scale σ = 2); error bars are standard deviation. **(D)** The PD map in a topo-VAE with σ = 2, the hyperparameter value which well approximates the recorded neuron topography (cf. S4 Fig for other values of length scale σ).

Having demonstrated similarity in the tuning statistics between modelled and recorded neurons, we next compared the spatial relationship of PDs in our model neurons to those in the monkey brain within the constraints of the 400 μm spacing of the recording electrodes (Fig 4). While neurons recorded at two adjacent electrodes corresponded to a distance that was substantially beyond the effective range of the Mexican hat distance function, many electrodes recorded data from more than one distinguishable neuron. The distance between these neurons was within the local neighbourhood of neurons in our model. We could thus compare the similarity of neural coding properties (specifically, the PDs) between neurons recorded on the same electrode to the similarity between neurons on different electrodes (cf. Fig 4A). In modelled neurons, same-electrode PD differences followed an exponential distribution, such that the probability that two neurons had similar (within 30 degrees) PDs was much higher than chance (p = 0.81; Fig 4C., blue histogram). Conversely, the probability that two neurons on the same electrode had nearly opposite PDs (between 150° and 180°) was very low (p = 0.032; Fig 4C orange histogram). These distributions were significantly different for nearby vs distant neurons (p<0.05; two-sided Wilcoxon signed-rank test). We then ran the same analysis on recorded neurons (Fig 4C) and found that there was no significant difference between the nearby PD difference distributions (orange) in modelled vs recorded neurons when *σ* = 2 (p > 0.05; Wilcoxon rank-sum test). Different choices of the neighbourhood hyperparameter, *σ*, led to differences in the exponential distributions of PD difference (S4B and S4C Fig). Small neighbourhood values (*σ* = 1, S4B and S4C Fig, left) led to more uniform distributions, whereas larger neighbourhood values (*σ* = 3, S4B and S4C Fig, right) led to a more exponential distribution, both of which were significantly different from the distributions observed in recorded neurons (p < 0.05; Wilcoxon rank-sum test). This would suggest that a lateral effect range of *σ* = 2 most accurately captures lateral interaction distances in proprioceptive cortex.

Given this result, we next use the topo-VAE model to directly predict the spatial organization of PD tuning across the proprioceptive cortex, which the 400um electrode spacing lacks the adequate spatial resolution to observe. The model predicts that PDs are clustered in blob-like structures of similar PDs with boundary regions where the PDs rotate smoothly toward neighbouring directions (Fig 4D). This arrangement is interrupted by small, sparsely spaced “pinwheel” regions, representing all PDs in a small neighbourhood, which is consistent with same-electrode similarity of Fig 4B and 4C.

**Fig 5:**
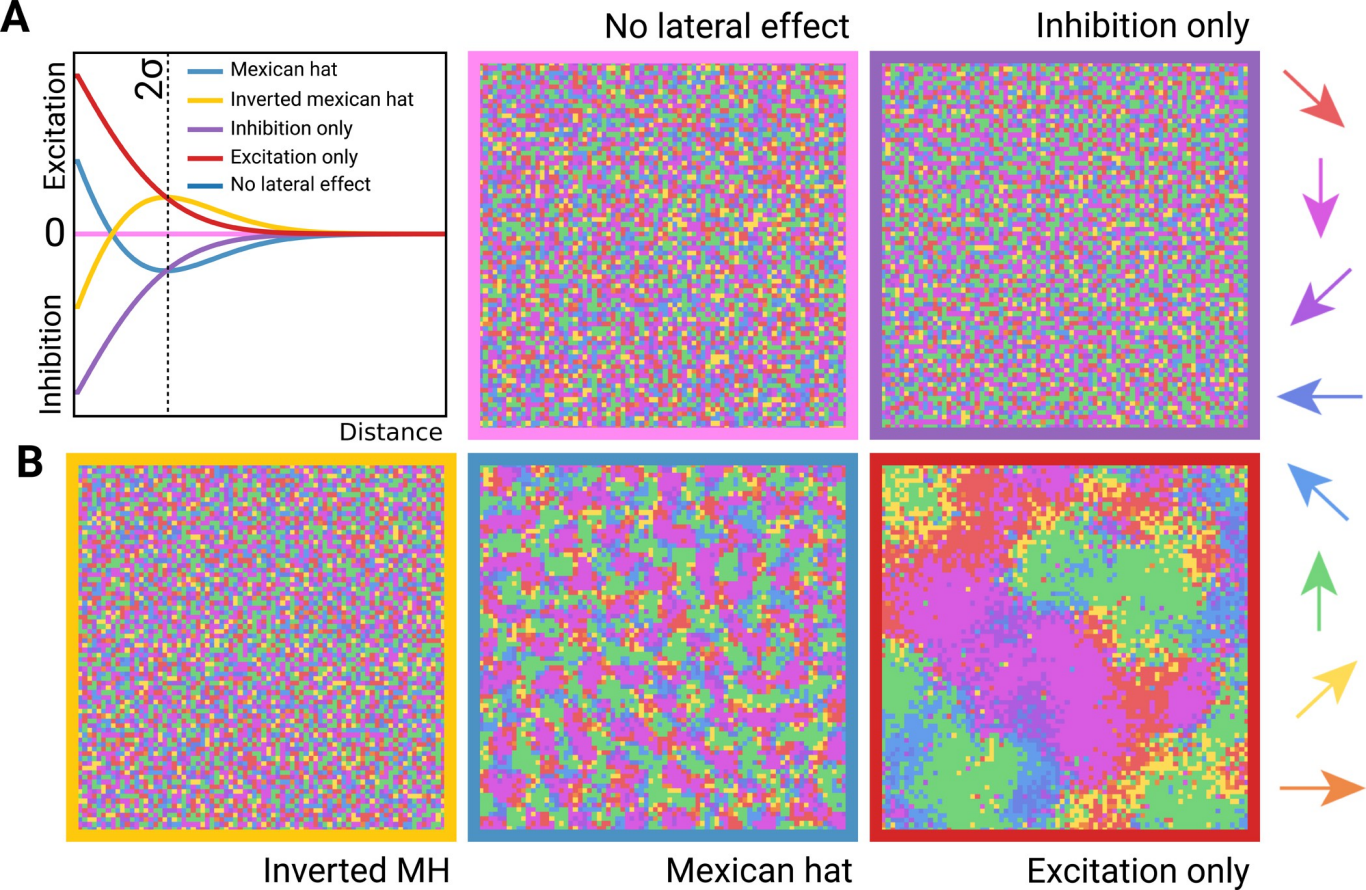
Mexican hat lateral effects are essential for reproducing spatial organisation. **(A)** Alternative lateral effect functions tested in control experiments, compared to the Mexican hat (blue line). **(B)** resulting PD maps for each function (colour matched to 5D).

Next, we examined which specific elements of the lateral interaction were important for reproducing the recorded data. We verified through model ablation the importance of the shape of the Mexican hat function (blue curve in Fig 5A), which determines the strength of excitatory and inhibitory connections as a function of distance between two neurons. We found no lateral effect function with a significant effect on input data reconstruction accuracy when compared to a model with no lateral effects (P<0.005; ANOVA). Flipping the function upside down (i.e., short range inhibition and intermediate range excitation, yellow curve in Fig 5A) eliminated the obvious topographic structure (Fig 5B, yellow box). Similarly, removing either the excitatory (purple curve in Fig 5A) or inhibitory component (red curve in Fig 5A) produced results unlike the localised topographic structure of our primary model against which the recorded data was validated (Fig 5B, purple and red boxes, respectively), suggesting that the specific Mexican hat shape was important for reproducing biological results.

We also investigate whether this topographic organisation emerges when the model is trained on shuffled kinematic data, to determine if the inherent structure of movement data (e.g. the covariance between joints and the constraints due to musculoskeletal geometry) contributes to the topographic organisation. We find that, while some level of organisation remains (S5A Fig), it is less pronounced (S5B Fig), suggesting that the structure of natural movement shapes topographic organisation in conjunction with lateral effects.

**Fig 6:**
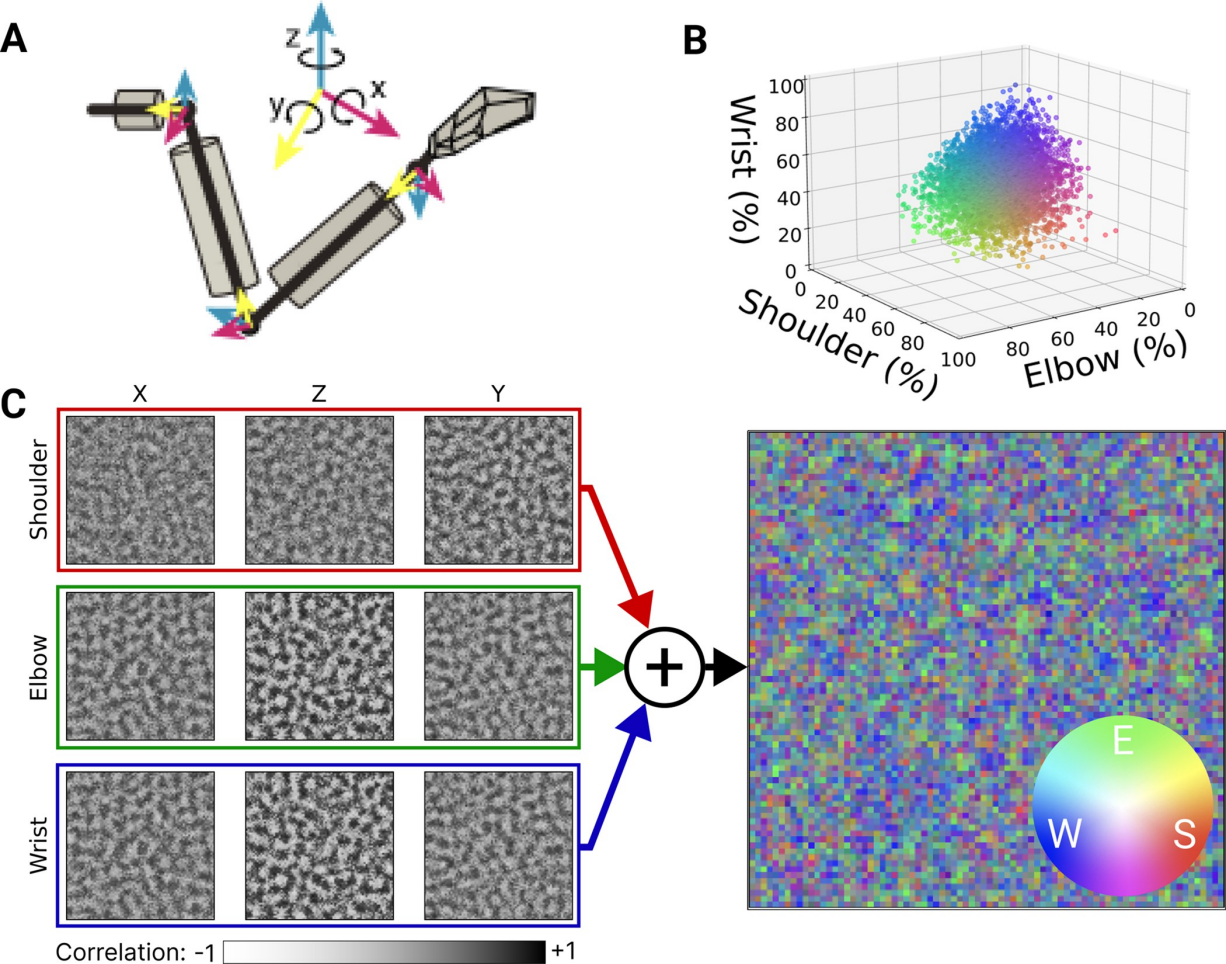
Sensitivity of latent layer neurons in topo-VAE to different input dimensions. **(A)** Joint angle axes, 3 per joint. **(B)** 3D plot showing the relative sensitivity of each neuron to joint inputs. Sensitivity is defined as the sum of correlations across X/Z/Y axes of all joints for a given neuron (colour normalised by maximum values per joint) (n = 6400). **(C)** Correlations between each dimension of input joint motion and neural response. (**D)** Joint sensitivity map where each pixel represents a neuron and the pixels RGB values (colour wheel inset) are reflecting the correlation with shoulder (“S”), elbow (“E”), and wrist (“W”) angular velocity, respectively (colours normalised by maximum value across joints).

In addition to predicting the spatial organisation of proprioceptive coding, our model enables testable predictions about the representation of movements involving multiple joints (wrist, elbow, shoulder) across the neural population. To obtain a measure of how the 3 joints (Fig 6A) are encoded across the cortical surface relative to each other, we found the average correlation with firing rate for all the degrees of freedom of each joint. All joints were evenly represented across the whole population (Fig 6B), but no neurons correlated with only one joint. Many modelled neurons were correlated even with non-adjacent joints, e.g. elbow-wrist or even all three joints, possibly a result of the natural covariance patterns that exist between joints. For pairwise joint sensitivity comparisons see scatter plots (S6 Fig).

Fig 6C shows in more detail, the response strength of our cortical model neurons for individual degrees of freedom. The maps had a spatial pattern of ripple-like transitions between positive (white) and negative (black) correlations (Fig 6C), producing topographic clusters of neurons that are sensitive to particular joints (Fig 6D). The scale of these clusters approximately matched the that of the Mexican-hat lateral connection range. Regions of pure red, green, or blue in Fig 6D would correspond to neurons responsive to only shoulder, elbow, or wrist respectively. Instead, colours that are the result of blending two or more joints predominate.

## Discussion

Here, we combine natural proprioceptive stimuli with a novel model of neural coding and topographical organization of neurons in somatosensory cortex to help develop our understanding of proprioceptive coding and allowing us to paint a picture beyond that we can see through the restricted lens of neural recordings. Core to our approach was allowing the model to learn to represent the statistics of natural human movements rather than stereotyped movements, which under the efficient coding hypothesis is key to understanding sensory representations in the nervous system [27,37–39].

Our topo-VAE is designed to reflect information processing in cortex: we hypothesise that the cortex learns, through experience, an efficient representation of the somatosensory world which we replicate here as a deep autoencoder network. The network learns a nonlinear mapping from kinematic inputs into a “cortical” latent space of spiking neurons, which embodies a stochastic, generative model of movement-related neural activity. While VAEs have been used before to learn both single neuron and population level features from spike train data [40,41] a vanilla VAE cannot induce a topographic mapping on the data. Consequently, they cause nearby input variables (stimuli) to be represented in arbitrary locations across the latent space. By contrast, the latent space of a topo-VAE is arranged as a two-dimensional cortical surface, thereby creating a population of neurons on a square grid. This enables a lateral interaction term between neighbouring neurons to be added, that shapes cortical activity into local neighbourhoods, allowing it to learn cortical-like spatial structure as well as temporal single-neuron activity patterns, both of which we compare to recorded neural data.

Previous self-organising maps [23,42,43] operate on less biologically plausible grounds. For example, Kohonen-type maps operate on winner-takes-all, non-spiking activation (and consequently, their training updates synaptic connections in a winner-takes-all form as well) [43]. Therefore, only one neuron can ever be active for given a sensory stimulus in a Kohonen map, a property very unlike actual proprioceptive cortex. In contrast, any number of neurons in our topo-VAE can be active simultaneously (and consequently, all synaptic connections are updated as sensory information is processed). Likewise, Poisson GLMs consider only the input statistics when producing simulated neural responses. While it is possible to reproduce the coding properties of individual neurons from convergence of peripheral inputs to a GLM, those models can tell us little about the role of neighbouring cells in shaping receptive fields or neuroanatomy-neural function relationships in general. It is important to note that there would be alternative ways to introduce lateral effects in the latent space which might better reflect biological circuits than the loss term used here. For example, our current approach simply encourages nearby neurons to be active when driven by similar inputs, and distant neurons to be active for differing inputs. However, lateral effects in biological circuits involve causal excitatory and inhibitory interactions between neurons. Such interactions may be better modelled in a network with recurrent feedback, which would also enable the latent space to be conditioned on sequences of stimuli. Alternatively, conditional random fields [44,45] could capture this property without the need for recurrent feedback, by conditioning the probability of a neuron firing on both the input, and the state of neighbouring neurons (defined by an undirected graph similar to Ψ).

Emergent from our model, were neurons with tuning surfaces comparable to those of recorded neurons as monkeys performed the same centre-out task (Fig 2A and 2B). Furthermore, higher-level features of the modelled neurons, such as PD distributions and the distributions of tuning curve features, were comparable to recorded neurons, particularly when the model was constrained by lateral effects and trained on stimuli that reflected the natural distributions of arm movement (Figs 2 and 3). However, they were not captured perfectly (e.g. the PD and tuning geometry distributions of modelled neurons were still significantly different from those of recorded neurons, p<0.005; Kolmogorov-Smirnov test). Despite this, the current model is able provide multiple predictions about the nature of proprioceptive coding, one of which we partially validate with our limited neural data. Future work includes identifying additional constraints that can allow the model to better reproduce neural data from proprioceptive cortex.

The well-known somatosensory homunculus represents only the tactile component of somatosensation and its well-ordered map of the skin receptors. Since the human tactile homunculus has driven much of neuroscience’s intuition about somatosensory representations, it is tempting to hypothesise that proprioceptive representations might also be similarly structured. However, proprioception is driven not by receptors embedded in a “simple” two-dimensional sheet, but rather by different types of muscle receptors with varying dynamics, that span one, two, and even three joints. Thus, the expectation that proprioception and touch might share a similar homunculus may not be reasonable. Indeed, instead of a point-to-point mapping to the limb, in our model, we find structure akin to the pinwheels of the visual cortex, here representing hand movement direction instead of orientation selectivity. While the relatively large spacing of electrodes used to record neural data from area 2 does not allow us to confirm the pinwheel anatomical structure directly, there are signatures of it in the recordings, such as the fact that neurons recorded on a given electrode tend to have more similar PDs than those recorded on separate electrodes–a necessary, but not sufficient property for proprioceptive pinwheels.

Our model predicts not only the spatial organisation of tuning, but also the receptive fields of single neurons–although this prediction remains difficult to test with our currently available data. We find that modelled neurons encode combinations of joints, both adjacent (shoulder-elbow) and distant (shoulder-wrist). Such convergence in monkey somatosensory cortex has been observed for the hand [46,47] and must be present to some extent in the proximal arm, given its multi-articular muscles. Our model used only joint-based inputs (i.e., the kinematic state or pose of the arm) but knows nothing about the musculoskeletal mechanics of the limb (e.g., the fact that bi-articulate muscles span multiple joints), beyond what can be inferred from correlations between joint angles. Nonetheless, multi-joint coding emerged for most neurons in our model. The correlations between motion of different joints that we observe in the natural data are substantial (4 principal components explain 80% of the variance of the seven degrees of freedom of the arm) and are the result of three main factors: 1) the biomechanics of the body, including the way many muscles span multiple joints, 2) the way the brain controls movements and 3) the tasks performed. Arguably, task requirements drive a substantial amount of the correlations. By training the topo-VAE on a highly varied dataset of natural movement, we enable it to generalise from these natural tasks to planar centre-out movements. Using this same dataset, Ejaz and colleagues showed that the pairwise similarity of finger-specific fMRI activity patterns in human sensorimotor cortex was better explained by the correlation structure of hand movements than muscle activity [37]. Therefore, the emergence of multi-joint proprioceptive receptive fields may be a further example of the shaping of neural activity by higher-order features of movements [48], analogous to higher-order features of visual receptive fields, such as edges in V1 [22].

It is important to acknowledge that while the comparisons between modelled and recorded neurons are promising, it is largely a qualitative similarity, particularly at the level of population statistics. Understanding what further constraints might enable our model to better reproduce these features will be an important step in further elucidating the principles that shape proprioceptive representations in the brain. We also make the key assumption that the proprioceptive organizational principles captured by our topo-VAE model based on the kinematics of human movement would extend to monkeys. While this assumption seems to have largely held, there may still exist differences that contribute to the inconsistencies between our model and recorded neurons. This may be addressed in future work using musculoskeletal models [49,50] to identify upper limb kinematics that are inconsistent between macaques and humans and filter them from the training data.

Another key limitation of the current model is the use of only joint kinematics. Biological inputs to proprioceptive cortex are received from muscle spindles and Golgi tendon organs, which convey muscle length, velocity, and force information. An important future direction will be to test the topo-VAE on such inputs, whether they be recorded experimentally or inferred from existing kinematic data using musculoskeletal simulations. Furthermore, because of its accessibility with Utah multielectrode arrays, we compared our modelled neurons to those recorded in area 2 of the somatosensory cortex, an area that combines cutaneous and muscle information in single neurons. Area 3a, on the other hand, has only muscle-receptor inputs, and shares many features with the adjacent motor cortex. One might expect an even closer correspondence between our model and area 3.

Lastly, we would like to highlight the observation that, when trained on the stereotyped centre-out-data only, the topo-VAE predicted recorded neuron data more poorly than when it was trained on the richer natural movement data. We interpret this to reflect the efficient coding hypothesis [26], whereby a population of sensory neurons are optimised to code stimuli representative of those found in their natural environment. Such codes are well documented in the visual [22] and auditory systems [51]–here we provide evidence that this may also be the case for proprioceptive neurons. This points towards the importance of working with training data that matches the natural distribution of stimuli when modelling sensory neurons, as it suggests important features of the neural code (such as the complex receptive fields predicted by our model) will only be elucidated under these conditions. This observation may be of great importance for the many proprioceptive and motor neurophysiology experiments that have been conducted in highly constrained lab settings, settings that may not contain adequate ethologically relevant kinematic statistics to uncover the true coding of cortical neurons. Undertaking electrophysiological experiments with a broader repertoire of movements may affect proprioceptive neuroscience as much as the adoption of natural images did for understanding vision.

## Methods

### Ethics statement

All surgical and experimental procedures were fully consistent with the guide for the care and use of laboratory animals and approved by the institutional animal care and use committee of Northwestern University under protocol number IS00000367. All human participants provided written consent, and experiments were approved by Imperial College Research Ethics Committee under Reference No. 18IC3815.

### Variational autoencoder with topographic latent space

In the following we lay out the rationale for building our model and the model itself, the various forms of data we collected for model training and validation, and the validation methodology.

The topo-VAE model (cf. Fig 1D–1G) uses the autoencoder framework to model sensory representations. Autoencoders are a type of artificial neural network used to learn efficient encodings of unlabelled data [52]. We choose a variational autoencoder (VAE) [28] because we want the latent cortical layer to capture the stochasticity and variability inherent to neural representations [53]. Let $X=\left\{x_{1},\ldots ,x_{N}\right\},x_{n}\in {\mathbb{R}}^{D}$ be a set of *N* sensory stimuli consisting of *D* features, following the natural behaviour distribution *p*(*X*). A group of cortical neurons, arranged in a 2D grid, are activated by the sensory stimuli *X* and generate firing patterns $Z=\left\{z_{1},\ldots ,z_{N}\right\},z_{n}\in {\mathbb{R}}^{M\times M},\left(M^{2}\gg D\right)$. This relationship is captured by the conditional distribution *p*(*Z*│*X*), which is dependent on *p*(*X*), and therefore intractable. However, it is possible to approximate *p*(*Z*│*X*) through variational inference by optimising a more tractable distribution, *q*(*Z*), to minimise the evidence lower bound (ELBO), which can be derived from the Kullback-Leibler divergence between *q*(*Z*) and *p*(*Z*│*X*):

$$\begin{array}{l} D_{KL}[q(Z)|p(Z|X)]=\int q(Z)\log\frac{q(Z)}{p(Z|X)}dZ\\ =E_{q(Z)}\left[\log\frac{q(Z)}{p(Z|X)}\right] \end{array}$$ (1)

$$\begin{array}{l} =E_{q(Z)}[\log\;q(Z)]-E_{q(Z)}[\log\;p(Z,X)]+\log\;p(X)\\ \geq E_{q(Z)}[\log\;q(Z)]-E_{q(Z)}[\log\;p(Z,X)]=-ELBO \end{array}$$

By maximising the ELBO, we avoid having to compute *p*(*X*) while still guaranteeing that the *D*_*KL*_[*q*(*Z*)││*p*(*Z*│*X*)] is minimised, since:

$$\begin{array}{l} \log\;p(X)=E_{q(Z)}[\log\;p(Z,X)]-E_{q(Z)}[\log\;q(Z)]+D_{KL}[q(Z)|p(Z|X)]\\ =ELBO+D_{KL}[q(Z)|p(Z|X)] \end{array}$$ (2)

where log *p*(*x*) is a fixed quantity for a given *X*.

In the case of a VAE, *P*(*Z*│*X*) is approximated by an encoder network *q*_*θ*_(*Z*│*X*) and *P*(*Z*│*X*) by a decoder network *p_ϕ_*(*X*│*Z*), where θ and ϕ represent the trainable parameters of the encoder and the decoder, respectively. For a given data sample *x*_*n*_ the network is optimised according to the loss function:

$$L_{n}(\theta ,\phi )=-E_{z∼q_{\theta }\left(z|x_{n}\right)}\left[\log p_{\phi }\left(x_{n}|z\right)\right]+D_{KL}\left[q_{\theta }\left(z|x_{n}\right)|p(z)\right]$$ (3)

Where $-E_{z∼q_{\theta }\left(z|x_{n}\right)}\left[\log\;p_{\phi }\left(x_{n}|z\right)\right]$ is the negative log-likelihood of the nth data sample (the reconstruction loss), and *D*_*KL*_[*q*_*θ*_(*z*│*x*_*n*_)│*p*(*z*)] is a regularisation term that pushes *q*_*θ*_(*z*│*x*_*n*_) towards some prior distribution (which can also be thought of as enforcing an expected firing rate on the neurons in the latent space).

In a conventional VAE the encoder network parameterises a Gaussian latent distribution. However, we choose to have the encoder network parameterise a Bernoulli latent distribution, such that *z*_*n*_ is sampled from a distribution of {0,1}, thus constraining the model to a spike-based encoding scheme (which allows modelled neurons to be analysed with the same methods as those used for the recorded neural data.). This sampling step is made differentiable (enabling gradient estimation of network parameters) using the Gumbel-softmax trick [54]:

$$Z_{i}=\frac{\exp\left(\frac{\log\left(\pi_{i}\right)+g_{i}}{\tau }\right)}{\sum_{j=1}^{2}\exp\left(\frac{\log\left(\pi_{j}\right)+g_{j}}{\tau }\right)}$$ (4)

where *τ* is the softmax temperature, which causes *z*_*n*_ to become one-hot as *τ* approaches 0 (a value of 0.1 is used for this work), *g*_*i*_ is a random sample from the Gumbel distribution *G*(0,1), defined by the probability density function:

$$g(x)=e^{-\left(x+e^{-x}\right)}$$ (5)

and *π* = [*p* 1-*p*] is a vector defining the probability of a neuron spiking vs. not spiking, where *p* is set by the output of the encoder network for each neuron.

For the Bernoulli prior, values closer to 0.5 enforce greater noise in the spiking activity of latent neurons. We choose a Bernoulli prior of p(Z = 1│X) = 0.01, which was found to enforce sparse activity in the latent space without decreasing reconstruction quality (S7 Fig, top panel).

In a conventional VAE, latent neurons lack the spatial relationships that constrain neural codes in cortex. Given this, we add a simple organisational mechanism that links learning of neighbouring latent neurons, effectively implementing cortical lateral connectivity with short range excitation and longer-range inhibition, by adding an additional term to the loss function of the VAE (Eq 3). This term is constructed by first assigning a unique 2D position to each neuron in the latent space, defined by $\Omega =\left\{\omega_{1},\ldots ,\omega_{M}\right\},\omega_{i}=\left[x_{i},y_{i}\right]$, which arranges neurons in an *M* × *M* topographic map. We then define a function, *ψ*, to specify the desired activity relationship between neurons. The Mexican-hat function, which transitions with distance from excitation to inhibition before vanishing [55] is a natural choice for cortical neurons (Fig 1F and 1G). For two neurons with indices, *p*,*q* ∈[1,*M*], the function *ψ*(*p*,*q*) returns the desired lateral effect between these neurons:

$$\psi (p,q)=\left(1-\frac{d{\left(\omega_{p},\omega_{q}\right)}^{2}}{2\sigma^{2}}\right)e^{-\frac{d{\left(\omega_{p},\omega_{q}\right)}^{2}}{2\sigma^{2}}}$$ (6)

where d(ω_*p*_,ω_*q*_)^2^ represents the Euclidean distance between neurons with indices *p* and *q* in the cortical grid:

$$d\left(\omega_{p},\omega_{q}\right)=\sqrt{{\left(x_{p}-x_{q}\right)}^{2}+{\left(y_{p}-y_{q}\right)}^{2}}$$ (7)

and *σ* is a hyperparameter defining the common length scale of local excitation and intermediate-range inhibition. As shown in Fig 1G, the transition from maximum excitation to maximum inhibition spans a distance of 2*σ* and the lateral effect vanishes at about 4*σ*. This term forms the basis of the topography in the topo-VAE, enabling it to learn both receptive field tuning properties (in an unsupervised manner through deep feature learning) and establishes a topographic relationship between neurons.

To compare our modelling and recording results, we chose hyperparameters of the topographic map with consideration of 1) spatial densities of S1 neurons and 2) characteristics of the neural recording devices. The density of neurons in S1 is about 8M – 17M per cm^3^ [56,57] of which 70% to 90% are pyramidal cells [58]. The thickness of cortex varies from 1 mm to 4.5 mm [59,60]. The Utah electrode array has 100 microelectrodes arranged in a 10 x 10 configuration with 400 μm separation along each axis, thus spanning 3.6 mm x 3.6 mm of the cortex. We model neuronal anatomy as voxels or cubes, where the number of pyramidal cells contained within 1mm^3^ of cortex varies from 12,000 to 100,000, which means every 1 mm along the cortical surface crosses about 20–50 pyramidal cells (see Fig 1C). The electrode array therefore captures a grid of approximately 80 x 80 neurons (for an evaluation of how decreasing this grid size affects the reconstruction score of the learned encoding, see S8 Fig). This means the range of the lateral effect parameter *σ* translates to a 1–2 neuron spacing on our cortical model grid (assuming the radius of dendritic input to these cortical neurons of around 200 μm [61]). The precise value of *σ* was determined post-hoc using model selection by numerically sweeping through a set of values (*σ* = {1,2,3}) and selecting the value that best reproduced Fig 4C. It is important to note that Fig 4B and 4C were similar across all tested values of *σ* when the Mexican hat lateral effect was used, while no other lateral effects achieved this similarity at any value of *σ*.

The topographic property of the VAE arises from the lateral effect component of the loss function (Eq 6). As a control, we tested our model with no lateral effects (Fig 5A–5D), and with several different distance functions (Mexican hat, inverted Mexican hat, excitation only, inhibition only; Fig 5F and 5G). The inverted Mexican hat lateral effect is the additive inverse of the Mexican hat function (Eq 6). Excitation-only lateral effect is defined as:

$$\psi (p,q)=e^{-\frac{d{\left(\omega_{p},\omega_{q}\right)}^{2}}{2\sigma^{2}}}$$ (8)

and the inhibition-only lateral effect is the additive inverse of the excitation-only lateral effect (Eq 8).

From the perspective of probabilistic inference, this regularisation item is equivalent to amending the prior distribution *p*(*z*)) and modifies the target function as:

$$\begin{array}{l} L_{n}(\theta ,\phi )=-E_{z∼q_{\theta }\left(z|x_{n}\right)}\left[\log\;p_{\phi }\left(x_{n}|z\right)\right]+D_{KL}\left[q_{\theta }\left(z|x_{n}\right)|p^{*}(z)\right]+const\text{,}\\ p^{*}(z)=\frac{p(z)e^{\gamma \mathrm{Tr}\left(z^{T}\Psi_{z}\right)}}{B}, \end{array}$$ (9)

where the normalisation factor $B=\int p(z)e^{Tr\left(z^{T}\Psi_{z}\right)}dZ$ is a constant. In this form, both the firing sparsity and the lateral effect are expressed within the amended prior distribution p^*^(*z*) and the target function is maintained in a standard variational autoencoder framework.

The overall total loss function for optimising the topo-VAE model is therefore given by:

$$L_{n}(\theta ,\phi )=-E_{z∼q_{\theta }\left(z|x_{n}\right)}\left[\log\;p_{\phi }\left(x_{n}|z\right)\right]+D_{KL}\left[q_{\theta }\left(z|x_{n}\right)|p(z)\right]-\gamma E_{z∼q_{\theta }\left(z|x_{n}\right)}\left[Tr\left(z^{T}\Psi_{z}\right)\right]$$ (10)

where Ψ is a matrix representing the pairwise interactions between all the neuron indices in the range [1,*M*], with each element *Ψ_p,q_* = *ψ*(*p*,*q*), and *γ* weights the importance of the KL-lateral effect terms during optimisation. For the model we present in the results, γ was chosen such that the reconstruction score of the model was not decreased by excessive topographic organisation. If γ is set too low, no topography occurs. If γ is set too high, just a few neurons (that are closest neighbours of each other) will dominate the activity in the latent space across all input stimuli, resulting in poor reconstruction. Based on this, we found γ = 20 to be an ideal value (S7 Fig, bottom panel).

The encoder component of the model contains a fully connected hidden layer of size 500, with a tanh activation function, and a final linear readout layer with a sigmoid activation function. The reconstruction error of the model is measured using mean squared error. The decoder network is a linear mapping from neural activities *Z* to the reconstructions of the sensory stimuli $\hat{X}$.

The topo-VAE model was implemented in Python using PyTorch [62] and run on a GPU workstation. All models were trained for 100 epochs using the Adam optimiser with a learning rate of 0.001 and a batch size of 400 (found using a random search over hyperparameters to maximise reconstruction score).

### Human behaviour scenarios and natural movement data

We recorded full-body movements from 18 healthy right-handed participants in two experimental scenarios. In the natural behaviour scenario (see Figs 1B and S1), subjects performed unconstrained daily tasks in a working kitchen environment. As food preparation and feeding are universal behaviours, the only direction given to subjects was to prepare and eat an omelette. For this modelling work we used only the arm movement data (including wrist but not digits). The average recording time across subjects was 22 minutes. In the second movement scenario, a subject performed planar centre-out reaches in a 20x20 cm horizontal task space (see Fig 1C) to mimic the movement data for the monkey task. The horizontal task space was aligned 20 cm below the subject’s shoulder and centred on the mid-line at 30 cm forward of the chest.

Arm movements in both scenarios were recorded at 60 Hz by an XSENS 3D motion tracking suit, a full-body sensor network based on inertial sensors. We used biomechanical models and fusion algorithms (including calibration and validation) to estimate joint angles. Fig 1A shows the biomechanical structure and coordinate system used in this paper. Arm movement datasets were formatted as time series of angles between segments following the International Society of Biomechanics (ISB) Euler angle extractions [29] in a ZXY coordinate. For the elbow and the wrist, angular rotations of Z, X and Y represent flexion/extension, abduction/adduction, and internal/external rotation, respectively. During planar movements, we used optical tracking as well as the motion tracking suit for capturing the endpoint (hand) position on the task square. Data from the inertial and optical motion tracking systems were synchronised manually via cue-based movements before, during and after the recording period.

We computed angular velocity profiles from the recorded data and analysed the natural movement dataset *X*_*nat*_ and the planar movement dataset *X*_*pl*_. The first two principal components of the planar movements explained over 95% of the total variance, but only half of the variance of the natural movements. This reveals that joint velocities of planar movements are highly constrained, as expected, to a 2-dimensional subspace. We applied the manipulative complexity metric [63] to quantify the complexity of the movements, which is defined as:

$$C=1-\frac{2}{D-1}\sum_{j=1}^{D}\sum_{i=1}^{j}\left(VAF_{i}-\frac{1}{D}\right)$$ (11)

Where *VAF*_*i*_ is the variance captured by the i^th^ PC. Larger values of C indicate higher complexity and a value of *C* = 1 means that all PCs contribute equally to the total variance. The complexity of planar movements was 0.06, much lower than the natural movement complexity of 0.5. We defined the direction θ and speed *v* of planar movements in world coordinates: 90°/270° are respectively away from and towards the chest, 180°/0° are to the left and right.

At training time, we also pre-process the input data. The empirical distribution of joint angular velocities during movements in our tasks is symmetric, unimodal, with sharp peaks at zero and heavy tails towards large speeds. To ensure equal contributions to model optimisation, these features are standardised without mean centring (since they are already approximately zero-centred, and to preserve the correspondence of a zero-valued input to zero joint angular velocity). This pre-processing also improves the training efficiency and convergence of our model without breaking the spatial structure of natural movements. In addition, since the original kinematic data is sampled at a frequency of 60Hz, we subsample the training data at a depth of 1%, to remove redundancy and improve training times, without significant effects on the outcome of the model.

After training, we evaluate the converged model with data from the planar reaching task, which undergoes the same pre-processing steps as the training data.

### Model analysis

Preferred directions are one of the primary tuning properties used when comparing modelled and recorded neurons in this work. To calculate the preferred direction of a neuron (both modelled and recorded), we use a simple bootstrapping procedure. For each iteration, we draw random points from the dataset conforming to a uniform distribution of movement directions and fit Poisson generalised linear models (GLM) with angular velocity inputs to the firing rate for the sampled timepoints. For a given input *X* ‐ a *T* by 2 (number of velocity inputs) matrix ‐ the GLM model predicts a probability mass function of firing rates, *y*, according to:

$$p(y|x;\beta )=\frac{\lambda^{y}}{y!}e^{-\lambda },\lambda =e^{X\beta }$$ (12)

Sampling *y* from this distribution gives a *T* (number of time points) by *N* (number of neurons) matrix of firing rate estimates. *β* is a 2 by *N* matrix of encoding parameters, which is optimised using maximum likelihood estimation. The preferred direction is then calculated from the encoding vector of the GLM as:

$$PD_{i}=\tan^{-1}\left(\beta_{i_{y}},\beta_{i_{x}}\right)$$ (13)

In these equations, a bootstrapped PD estimate of neuron *i* is defined by $\beta_{i_{y}}$ and $\beta_{i_{x}}$, the GLM parameters for mapping hand velocity in the *y* and *x* directions to firing rate estimates. We take the circular mean of the PD estimates over all bootstrap iterations to find the PD for each neuron. This method of PD calculation is used for both recorded and modelled neurons.

To quantify the sensitivity of neurons to specific joints (Fig 6), we individually perturb each input feature of the model and measure the Pearson correlation between individual neurons in the latent space and that feature. Since each joint is represented by three input features (angular velocity in the Z, X, and Y axes), we use the mean of the absolute correlation across all three axes to quantify the sensitivity of a given neuron to a particular joint.

### nonhuman primate behaviour and data collection

We used a combination of previously recorded data in which three rhesus macaques performed a planar, centre-out reaching task while seated, using a two-link planar manipulandum. A cursor displayed on a monitor tracked the position of the manipulandum and provided visual feedback for the monkey as he reached for a target on-screen. The monkey moved the cursor to a central target in the workspace. After a random delay period, 1 of 8 targets spaced evenly in a circle around the central target appeared on the screen and the monkey moved the cursor toward it upon an audible ‘go’ cue. After placing the cursor in the target for a random hold time of 0–500 ms, the monkey received a liquid reward and returned the cursor to the central target. We used 6 experimental sessions across three monkeys; two sessions contain data that has been previously published [64], and the rest will be published alongside this study. All procedures were in accordance with the Guide for the Care and Use of Laboratory Animals and were approved by the institutional animal care and use committee of Northwestern University under protocol #IS00000367.

Once a monkey was trained on the experimental apparatus, a 96-channel microelectrode array with 1 mm iridium-oxide coated electrodes (Blackrock Microsystems, Inc.) was pneumatically inserted in Brodmann’s area 2, near the intraparietal sulcus [64]. The implantation site was chosen to avoid cerebral vasculature and maximise proximal arm representation. All surgery was performed under isoflurane gas anaesthesia (1–2 percent) except during intraoperative recording to identify the arm representations, when the monkey was transitioned to a mixture of <0.5% isoflurane and remifentanil (0.4 ug/kg/min).

The data were recorded from the microelectrode array using the Cerebus multichannel data recording system (Blackrock Microsystems, Inc.). Thresholded waveforms and timing of behavioural task events were synchronised and recorded for offline analyses. The position of the handle was recorded at 1kHz. We discriminated single neurons using Offline Sorter (Plexon, Inc., Dallas TX). 383 recorded neurons were found to be suitable for subsequent analysis.

## Supporting information

S1 Fig Natural kinematics from behaviour in daily life. **(A)** Examples of natural behaviours contained in the dataset, as seen through egocentric video. **(B)** 9-dimensional angular joint velocity time series of ZXY joint axes (blue/green/orange lines, respectively) for shoulder, elbow, and wrist joint. Red lines correspond to the frames shown in (A).
(SVG)

S2 Fig Additional tuning comparison of modelled and recorded neurons. Tuning maps from example neurons observed in the latent layer of the topo-VAE under planar movements (top) and example area 2 neurons during planar movements (bottom), matched to the example modelled neurons. Note that these are examples from around the 50^th^ percentile of match quality.
(SVG)

S3 Fig Bimodality of PD distributions. Fit quality (AIC & BIC) of Gaussian mixture model fits with a varying number of modes for the preferred direction (PD) distributions of **(A)** Modelled neurons and **(B)** Recorded neurons. Note that model quality continues to improve (decreasing AIC/BIC value) with number of modes, however the most significant decrease occurs between 1 and 2 modes, while remaining improvements are small. This suggests the PD distributions of both modelled and recorded neurons are well captured by a bimodal distribution.
(SVG)

S4 Fig Effect of σ on cortical topography. **(A)** Distribution of PD difference for same electrode and different electrode comparisons in recorded neurons and **(B)** modelled neurons, with σ = 1, 2, and 3 shown on the left, middle, and right, respectively (n = 6400). Standard deviation across 10 models with different weight initialisations is indicated by a black bar for each bin. **(C)** PD maps for σ = 1, 2, and 3 shown on the left, middle, and right, respectively. A visualisation of the lateral effect under σ is also shown inset for each map.
(SVG)

S5 Fig Effect of shuffled training data on cortical topography. Topographic organisation of PDs when the topo-VAE is trained on **(A)** Shuffled kinematic data and **(B)** The original kinematic data (same as Fig 4D of the main paper).
(SVG)

S6 Fig Tuning of modelled neurons to arm joints. Tuning is quantified by the correlations between the activity of a neuron and joint angle velocity inputs from the shoulder, wrist, and elbow (summed across Z/X/Y planes for each joint). The sum of correlations across joints is then normalised such that a neuron with equal elbow, shoulder and wrist tuning would be positioned at 33.33% for each joint in the above plots. Each plot shows the relative tuning of each neuron (one data point) to joint inputs for the following pairs: **(A)** Elbow vs Shoulder, **(B)** Wrist vs Elbow, and **(C)** Wrist vs Shoulder. The colour of each dot is set by using their x, y, and z location in the plot to drive their respective red, green, and blue colour channels. The 3D plot of neurons for wrist, elbow and shoulder together are shown in Fig 6 in the main text.
(PNG)

S7 Fig Hyperparameter effects on tuning maps and reconstruction. Effect of changing the firing rate prior (top) and lateral effect cost weighting (bottom) on the tuning maps in the latent space (left hand side) and reconstruction scores (R2 score, natural-trained model reconstruction planar reaching data) (right hand side). Red boxes indicate the hyperparameter values chosen in the model used in the results.
(SVG)

S8 Fig Effect of latent size on reconstruction quality. R2 score (y-axis) of the reconstruction of kinematic data from planar reaching movements in networks trained on kinematic data from natural movement with varying latent space sizes (x-axis).
(SVG)

## References

[1] Todorov E, Jordan MI. Optimal feedback control as a theory of motor coordination. Nat Neurosci. 2002. doi: 10.1038/nn963 12404008

[2] Scott SH. Optimal feedback control and the neural basis of volitional motor control. Nature Reviews Neuroscience. 2004. doi: 10.1038/nrn1427 15208695

[3] Tuthill JC, Azim E. Proprioception. Current Biology. 2018. doi: 10.1016/j.cub.2018.01.064 29510103

[4] Sainburg RL, Poizner H, Ghez C. Loss of proprioception produces deficits in interjoint coordination. J Neurophysiol. 1993. doi: 10.1152/jn.1993.70.5.2136 8294975 PMC10710694

[5] Flesher SN, Downey JE, Weiss JM, Hughes CL, Herrera AJ, Tyler-Kabara EC, et al. A brain-computer interface that evokes tactile sensations improves robotic arm control. Science (80-). 2021. doi: 10.1126/science.abd0380 34016775 PMC8715714

[6] Salas MA, Bashford L, Kellis S, Jafari M, Jo H, Kramer D, et al. Proprioceptive and cutaneous sensations in humans elicited by intracortical microstimulation. Elife. 2018. doi: 10.7554/eLife.32904 29633714 PMC5896877

[7] EZ H, EL G, WD M, R A, G FB-V, BC H, et al. Reconnecting the Hand and Arm to the Brain: Efficacy of Neural Interfaces for Sensorimotor Restoration After Tetraplegia. Neurosurgery. 2024;94: 864–74. doi: 10.1007/978-3-319-47313-0_20 PMC12245341 37982637

[8] Tomlinson T, Miller LE. Toward a proprioceptive neural interface that mimics natural cortical activity. Advances in Experimental Medicine and Biology. 2016. doi: 10.1007/978-3-319-47313-0_20 28035576 PMC5452683

[9] Faisal AA. Putting touch into action. Science (80-). 2021. doi: 10.1126/science.abi7262 34016768

[10] Formento E, Minassian K, Wagner F, Mignardot JB, Le Goff-Mignardot CG, Rowald A, et al. Electrical spinal cord stimulation must preserve proprioception to enable locomotion in humans with spinal cord injury. Nat Neurosci. 2018. doi: 10.1038/s41593-018-0262-6 30382196 PMC6268129

[11] Weber DJ, Friesen R, Miller LE. Interfacing the somatosensory system to restore touch and Proprioception: Essential considerations. Journal of Motor Behavior. 2012. doi: 10.1080/00222895.2012.735283 23237464

[12] Blum KP, Versteeg C, Sombeck J, Chowdhury RH, Miller LE. Proprioception: a sense to facilitate action. Somatosensory Feedback for Neuroprosthetics. 2021. doi: 10.1016/b978-0-12-822828-9.00017-4

[13] Penfield W, Boldrey E. Somatic motor and sensory representation in the cerebral cortex of man as studied by electrical stimulation. Brain. 1937. doi: 10.1093/brain/60.4.389

[14] Weber DJ, London BM, Hokanson JA, Ayers CA, Gaunt RA, Torres RR, et al. Limb-state information encoded by peripheral and central somatosensory neurons: Implications for an afferent interface. IEEE Trans Neural Syst Rehabil Eng. 2011. doi: 10.1109/TNSRE.2011.2163145 21878419 PMC3694199

[15] Rossi LF, Harris KD, Carandini M. Spatial connectivity matches direction selectivity in visual cortex. Nature. 2020. doi: 10.1038/s41586-020-2894-4 33177719 PMC7116721

[16] Killebrew JH, Bensmaïa SJ, Dammann JF, Denchev P, Hsiao SS, Craig JC, et al. A dense array stimulator to generate arbitrary spatio-temporal tactile stimuli. J Neurosci Methods. 2007. doi: 10.1016/j.jneumeth.2006.10.012 17134760 PMC1851669

[17] Mohsenzadeh Y, Mullin C, Lahner B, Oliva A. Emergence of Visual Center-Periphery Spatial Organization in Deep Convolutional Neural Networks. Sci Rep. 2020. doi: 10.1038/s41598-020-61409-0 32170209 PMC7070097

[18] Stringer C, Pachitariu M, Steinmetz N, Carandini M, Harris KD. High-dimensional geometry of population responses in visual cortex. Nature. 2019. doi: 10.1038/s41586-019-1346-5 31243367 PMC6642054

[19] Pehlevan C, Genkin A, Chklovskii DB. A clustering neural network model of insect olfaction. Conference Record of 51st Asilomar Conference on Signals, Systems and Computers, ACSSC 2017. 2018. doi: 10.1109/ACSSC.2017.8335410

[20] Sandbrink KJ, Mamidanna P, Michaelis C, Bethge M, Mathis MW, Mathis A. Contrasting action and posture coding with hierarchical deep neural network models of proprioception. Elife. 2023. doi: 10.7554/eLife.81499 37254843 PMC10361732

[21] Vargas AM, Bisi A, Chiappa A, Versteeg C, Miller L, Mathis A. Task-driven neural network models predict neural dynamics of proprioception. Cell. 2024. 10.1016/j.cell.2024.02.036 38518772

[22] Olshausen BA, Field DJ. Sparse coding with an overcomplete basis set: A strategy employed by V1? Vision Res. 1997. doi: 10.1016/S0042-6989(97)00169-7 9425546

[23] Obermayer K, Blasdel GG. Geometry of orientation and ocular dominance columns in monkey striate cortex. J Neurosci. 1993. doi: 10.1523/JNEUROSCI.13-10-04114.1993 8410181 PMC6576395

[24] Marr D. Vision: a computational investigation into the human representation and processing of visual information. Vis a Comput Investig into Hum Represent Process Vis information. 1982. doi: 10.1016/0022-2496(83)90030-5

[25] Linsker R. Self-Organization in a Perceptual Network. Computer (Long Beach Calif). 1988. doi: 10.1109/2.36

[26] Barlow HB. Possible Principles Underlying the Transformations of Sensory Messages. Sensory Communication. 2013. doi: 10.7551/mitpress/9780262518420.003.0013

[27] Laughlin S. A simple coding procedure enhances a neuron’s information capacity. Zeitschrift fur Naturforschung ‐ Section C Journal of Biosciences. 1981. doi: 10.1515/znc-1981-9-1040 7303823

[28] Kingma DP, Welling M. Auto-encoding variational bayes. 2nd International Conference on Learning Representations, ICLR 2014 ‐ Conference Track Proceedings. 2014.

[29] Wu G, Van Der Helm FCT, Veeger HEJ, Makhsous M, Van Roy P, Anglin C, et al. ISB recommendation on definitions of joint coordinate systems of various joints for the reporting of human joint motion ‐ Part II: Shoulder, elbow, wrist and hand. J Biomech. 2005. doi: 10.1016/j.jbiomech.2004.05.042 15844264

[30] Sterling P, Laughlin S. Principles of neural design. Principles of Neural Design. 2015. doi: 10.7551/mitpress/9780262028707.001.0001

[31] Bullmore E, Sporns O. The economy of brain network organization. Nature Reviews Neuroscience. 2012. doi: 10.1038/nrn3214 22498897

[32] Chen BL, Hall DH, Chklovskii DB. Wiring optimization can relate neuronal structure and function. Proc Natl Acad Sci U S A. 2006. doi: 10.1073/pnas.0506806103 16537428 PMC1550972

[33] Jacobs RA, Jordan MI. Computational consequences of a bias toward short connections. J Cogn Neurosci. 1992. doi: 10.1162/jocn.1992.4.4.323 23968126

[34] Christel MI, Billard A. Comparison between macaques’ and humans’ kinematics of prehension: The role of morphological differences and control mechanisms. Behav Brain Res. 2002. doi: 10.1016/s0166-4328(01)00372-2 11844584

[35] Kurtzer I, Herter TM, Scott SH. Nonuniform distribution of reach-related and torque-related activity in upper arm muscles and neurons of primary motor cortex. J Neurophysiol. 2006. doi: 10.1152/jn.00110.2006 17005623

[36] Herter TM, Kurtzer I, Cabel DW, Haunts KA, Scott SH. Characterization of torque-related activity in primary motor cortex during a multijoint postural task. J Neurophysiol. 2007. doi: 10.1152/jn.00757.2006 17267758

[37] Ejaz N, Hamada M, Diedrichsen J. Hand use predicts the structure of representations in sensorimotor cortex. Nat Neurosci. 2015. doi: 10.1038/nn.4038 26030847

[38] Simoncelli EP, Olshausen BA. Natural image statistics and neural representation. Annual Review of Neuroscience. 2001. doi: 10.1146/annurev.neuro.24.1.1193 11520932

[39] Ganguli D, Simoncelli EP. Efficient sensory encoding and Bayesian inference with heterogeneous neural populations. Neural Comput. 2014. doi: 10.1162/NECO_a_00638 25058702 PMC4167880

[40] Wu A, Roy NA, Keeley S, Pillow JW. Gaussian process based nonlinear latent structure discovery in multivariate spike train data. Advances in Neural Information Processing Systems. 2017. doi: 10.6080/K0NK3BZJ 31244512 PMC6594561

[41] Higgins I, Chang L, Langston V, Hassabis D, Summerfield C, Tsao D, et al. Unsupervised deep learning identifies semantic disentanglement in single inferotemporal face patch neurons. Nat Commun. 2021. doi: 10.1038/s41467-021-26751-5 34753913 PMC8578601

[42] Aflalo TN, Graziano MSA. Possible origins of the complex topographic organization of motor cortex: reduction of a multidimensional space onto a two-dimensional array. J Neurosci. 2006. doi: 10.1523/JNEUROSCI.0768-06.2006 16763036 PMC6675193

[43] Kohonen T. Self-organized formation of topologically correct feature maps. Biol Cybern. 1982. doi: 10.1007/BF00337288

[44] Sutton C, McCallum A. An introduction to conditional random fields. Found Trends Mach Learn. 2011. doi: 10.1561/2200000013

[45] Fu Y, Tan C, Bi B, Chen M, Feng Y, Rush AM. Latent template induction with gumbel-CRFs. Advances in Neural Information Processing Systems. 2020.

[46] Costanzo RM, Gardner EP. Multiple-joint neurons in somatosensory cortex of awake monkeys. Brain Res. 1981. doi: 10.1016/0006-8993(81)91197-5 7237174

[47] Warren JP, Santello M, Tillery SI. Effects of fusion between tactile and proprioceptive inputs on tactile perception. PLoS One. 2011. doi: 10.1371/journal.pone.0018073 21464943 PMC3064587

[48] Thomik AAC, Fenske S, Faisal AA. Towards sparse coding of natural movements for neuroprosthetics and brain-machine interfaces. International IEEE/EMBS Conference on Neural Engineering, NER. 2015. doi: 10.1109/NER.2015.7146780

[49] Chan SS, Moran DW. Computational model of a primate arm: From hand position to joint angles, joint torques and muscle forces. J Neural Eng. 2006. doi: 10.1088/1741-2560/3/4/010 17124337

[50] Holzbaur KRS, Murray WM, Delp SL. A model of the upper extremity for simulating musculoskeletal surgery and analyzing neuromuscular control. Ann Biomed Eng. 2005. doi: 10.1007/s10439-005-3320-7 16078622

[51] Lewicki MS. Efficient coding of natural sounds. Nat Neurosci. 2002. doi: 10.1038/nn831 11896400

[52] Hinton GE, Salakhutdinov RR. Reducing the dimensionality of data with neural networks. Science (80-). 2006. doi: 10.1126/science.1127647 16873662

[53] Orbán G, Berkes P, Fiser J, Lengyel M. Neural Variability and Sampling-Based Probabilistic Representations in the Visual Cortex. Neuron. 2016. doi: 10.1016/j.neuron.2016.09.038 27764674 PMC5077700

[54] Jang E, Gu S, Poole B. Categorical reparameterization with gumbel-softmax. 5th International Conference on Learning Representations, ICLR 2017 ‐ Conference Track Proceedings. 2017.

[55] Amari S ichi. Dynamics of pattern formation in lateral-inhibition type neural fields. Biol Cybern. 1977. doi:10.1007/BF00337259 10.1007/BF00337259 911931

[56] Turner EC, Young NA, Reed JL, Collins CE, Flaherty DK, Gabi M, et al. Distributions of Cells and Neurons across the Cortical Sheet in Old World Macaques. Brain Behav Evol. 2016. doi: 10.1159/000446762 27547956

[57] Collins CE, Turner EC, Sawyer EK, Reed JL, Young NA, Flaherty DK, et al. Cortical cell and neuron density estimates in one chimpanzee hemisphere. Proc Natl Acad Sci U S A. 2016. doi: 10.1073/pnas.1524208113 26729880 PMC4725503

[58] Kaas JH. Evolution of the neocortex. Current Biology. 2006. doi: 10.1016/j.cub.2006.09.057 17084684

[59] Fischl B, Dale AM. Measuring the thickness of the human cerebral cortex from magnetic resonance images. Proc Natl Acad Sci U S A. 2000. doi: 10.1073/pnas.200033797 10984517 PMC27146

[60] Wagstyl K, Ronan L, Goodyer IM, Fletcher PC. Cortical thickness gradients in structural hierarchies. Neuroimage. 2015. doi: 10.1016/j.neuroimage.2015.02.036 25725468 PMC4401442

[61] Braitenberg V, Schüz A. Cortex: Statistics and Geometry of Neuronal Connectivity. Cortex: Statistics and Geometry of Neuronal Connectivity. 1998. doi: 10.1007/978-3-662-03733-1

[62] Paszke A, Gross S, Massa F, Lerer A, Bradbury J, Chanan G, et al. PyTorch: An imperative style, high-performance deep learning library. Advances in Neural Information Processing Systems. 2019.

[63] Belić JJ, Faisal AA. Decoding of human hand actions to handle missing limbs in neuroprosthetics. Front Comput Neurosci. 2015. doi: 10.3389/fncom.2015.00027 25767447 PMC4341559

[64] Chowdhury RH, Glaser JI, Miller LE. Area 2 of primary somatosensory cortex encodes kinematics of the whole arm. Elife. 2020. doi: 10.7554/eLife.48198 31971510 PMC6977965

