# Supplementary figures and images for: Predicting proprioceptive cortical anatomy and neural coding with topographic autoencoders
Source: PLoS Comput Biol. 2024 Dec 4;20(12):e1012614. doi: 10.1371/journal.pcbi.1012614 (PMC11649110; doi:10.1371/journal.pcbi.1012614)

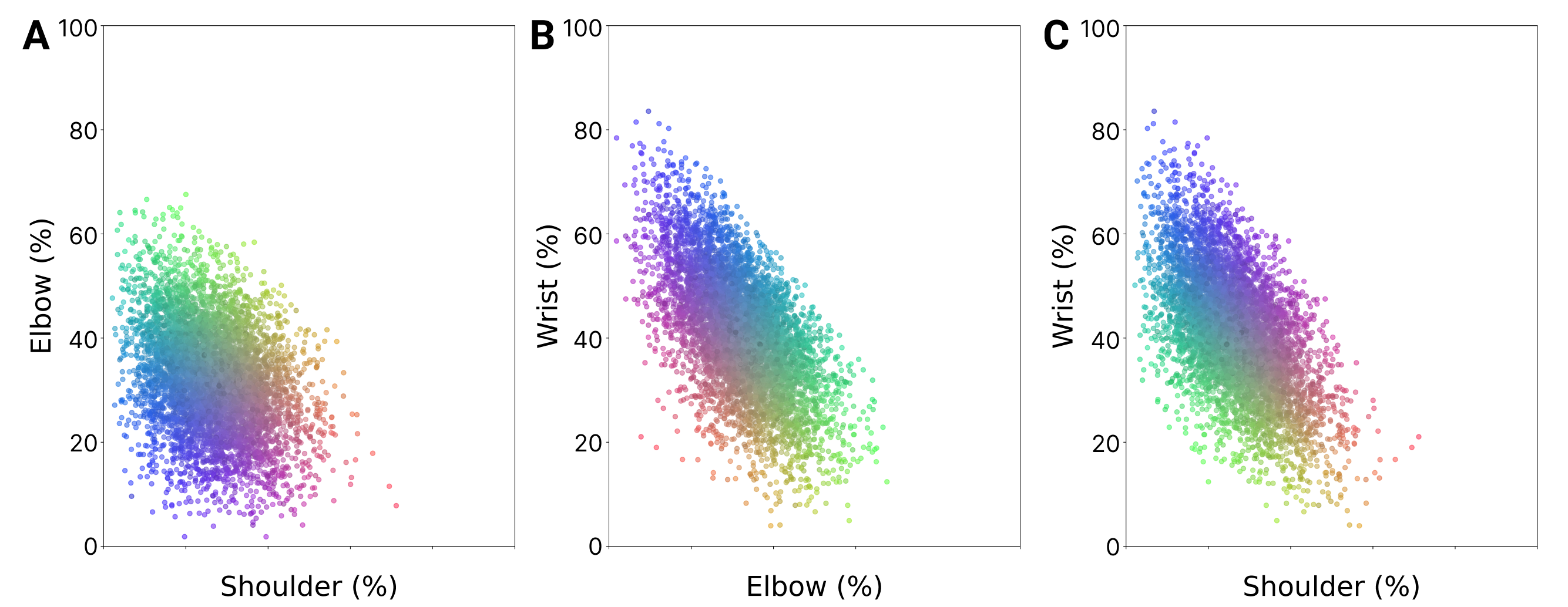

Supplement: S6 Fig — Tuning is quantified by the correlations between the activity of a neuron and joint angle velocity inputs from the shoulder, wrist, and elbow (summed across Z/X/Y planes for each joint). The sum of correlations across joints is then normalised such that a neuron with equal elbow, shoulder and wrist tuning would be positioned at 33.33% for each joint in the above plots. Each plot shows the relative tuning of each neuron (one data point) to joint inputs for the following pairs: (A) Elbow vs Shoulder, (B) Wrist vs Elbow, and (C) Wrist vs Shoulder. The colour of each dot is set by using their x, y, and z location in the plot to drive their respective red, green, and blue colour channels. The 3D plot of neurons for wrist, elbow and shoulder together are shown in Fig 6 in the main text. (PNG) [file pcbi.1012614.s006.png]
